# Plasmid Dissemination and Selection of a Multidrug-Resistant Klebsiella pneumoniae Strain during Transplant-Associated Antibiotic Therapy

**DOI:** 10.1128/mBio.00652-19

**Published:** 2019-10-08

**Authors:** Sean Conlan, Anna F. Lau, Clay Deming, Christine D. Spalding, ShihQueen Lee-Lin, Pamela J. Thomas, Morgan Park, John P. Dekker, Karen M. Frank, Tara N. Palmore, Julia A. Segre

**Affiliations:** a National Human Genome Research Institute, Bethesda, Maryland, USA; b National Institutes of Health Clinical Center, Bethesda, Maryland, USA; c National Institutes of Health Intramural Sequencing Center (NISC), Rockville, Maryland, USA; d National Institute of Allergy and Infectious Diseases, Bethesda, Maryland, USA; Louis Stokes Veterans Affairs Medical Center

**Keywords:** *Enterobacteriaceae*, HGT, *Klebsiella*, antibiotic resistance, carbapenems, genomes, plasmids

## Abstract

Antibiotics, which are used both to prevent and to treat infections, are a mainstay therapy for lifesaving procedures such as transplantation. For this reason, and many others, increased antibiotic resistance among human-associated pathogens, such as the carbapenem-resistant *Enterobacteriaceae* species, is of grave concern. In this study, we report on a hematopoietic stem cell transplant recipient in whom cultures detected the emergence of carbapenem resistance and spread across five strains of bacteria that persisted for over a year. Carbapenem resistance in Citrobacter freundii, Enterobacter cloacae, Klebsiella aerogenes, and Klebsiella pneumoniae was linked to a pair of plasmids, each carrying the Klebsiella pneumoniae carbapenemase gene (*bla*_KPC_). Surveillance cultures identified a carbapenem-susceptible strain of Citrobacter freundii that may have become resistant through horizontal gene transfer of these plasmids. Selection of a multidrug-resistant Klebsiella pneumoniae strain was also detected following combination antibiotic therapy. Here we report a plasmid carrying the *bla*_KPC_ gene with broad host range that poses the additional threat of spreading to endogenous members of the human gut microbiome.

## Abstract (precis)

Antibiotic-resistant bacteria are a serious threat to medically fragile patient populations. The spread of antibiotic resistance through plasmid-mediated mechanisms is of grave concern as it can lead to the conversion of endogenous patient-associated strains to difficult-to-treat pathogens.

## INTRODUCTION

The increase in carbapenem resistance among *Enterobacteriaceae* species is a worldwide threat (1, 2). Horizontal gene transfer (HGT) plays an important role in the spread of antibiotic resistance genes in clinical settings. Plasmid-borne resistance genes can turn infections with common organisms, like Escherichia coli into serious complications. Although guidelines to prevent transmission of carbapenem-resistant *Enterobacteriaceae* (CRE) exist (https://www.cdc.gov/hai/organisms/cre/cre-toolkit/index.html), they do not explicitly take into account HGT, which may complicate health care facility surveillance. Genomic studies like this one, and those conducted at other health care facilities (3–5), are critical to our understanding of plasmid-mediated antibiotic resistance and to improving local and global sequence databases. Here we investigate an exceptional case of a pair of plasmids that are widely distributed across isolates from a single patient, using both whole-genome sequencing (WGS) and metagenomic sequencing. We have combined treatment data, WGS, and metagenomics to explore the distribution and persistence of carbapenem resistance plasmids and changes to the microbiome associated with this patient’s antibiotic chemotherapy.

## RESULTS

### Case history.

**FIG 1:**
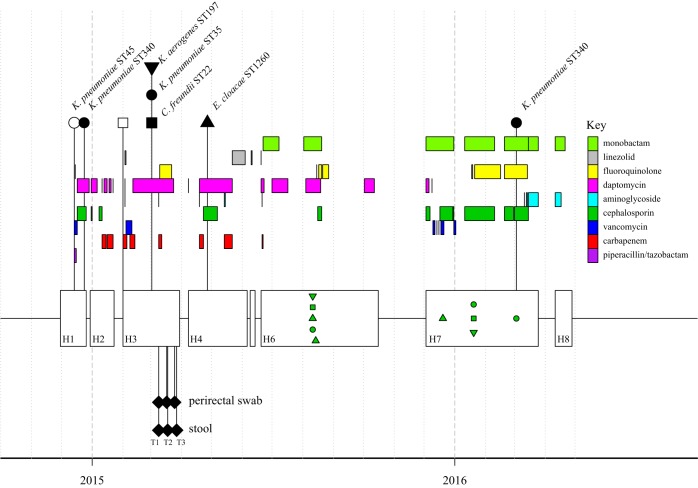
Time line of sequenced isolates, metagenomic samples, and antibiotic courses. Hospitalizations are shown as unfilled boxes (H1 to H8); the HSCT was performed during the first hospitalization. Antibiotic courses are indicated by colored bars; each antibiotic class is shown as a horizontal track. Sequenced isolates are shown at the top and connected by lines. Carbapenemase-producing isolates are shown as filled symbols. Isolates used for plasmid-specific PCR are indicated on the time line as green symbols; all were positive for one or both *bla*_KPC_ plasmids. Symbol shape represents the bacterial species: Klebsiella pneumoniae, circles; Klebsiella aerogenes, inverted triangles, Enterobacter cloacae, triangles; Citrobacter freundii, squares. A total of 88 carbapenem-resistant *Enterobacteriaceae* were collected over 17 months. Metagenomic samples are shown below the hospitalizations as filled diamonds.

In 2014, a patient entered care at the National Institutes of Health (NIH) Clinical Center for management of a hematologic malignancy. From December 2014 through April 2016, this patient was admitted on eight separate occasions, ranging in duration from 6 to 119 days. During the first admission (H1), the patient underwent a hematopoietic stem cell transplantation (HSCT) and started immunosuppressive therapy and supportive antibiotic therapy (Fig. 1). During that time, a carbapenem-susceptible Klebsiella pneumoniae strain of sequence type 45 (ST45) was cultured from blood and treated with piperacillin-tazobactam followed by ceftazidime. Ten days later, a carbapenemase-producing, *bla*_KPC_-positive K. pneumoniae strain (ST340) was cultured from an abscess. The patient continued to test positive for carbapenemase-producing K. pneumoniae across the patient’s remaining hospitalizations. In addition, surveillance and clinical cultures positive for *Enterobacteriaceae*, *Pseudomonas*, *Staphylococcus*, and *Streptococcus* spp. were detected (see Table S1 in the supplemental material). In March, during the patient’s third hospitalization, 68 days after the first *bla*_KPC_-positive K. pneumoniae strain was cultured, three *bla*_KPC_-positive *Enterobacteriaceae* species grew from a single perirectal surveillance culture: K. pneumoniae (ST35), Klebsiella aerogenes (ST197), and Citrobacter freundii (ST22). The patient continued to have *bla*_KPC_-positive cultures through 2016.

10.1128/mBio.00652-19.3 TABLE S1 Patient culture data. All cultures, including no-growth cultures, are shown in order to place the isolates described in this study in context. Antibiotic susceptibility results are provided only for Gram-negative rods. Susceptibility testing was performed following Clinical and Laboratory Standards Institute guidelines as described in Materials and Methods. Amox/Clav, amoxicillin-clavulanic acid; Pip/Tazo, piperacillin-tazobactam; Trim/Sulfa, trimethoprim-sulfamethoxazole. Download Table S1, XLSX file, 0.1 MB. Copyright © 2019 Conlan et al. 2019 Conlan et al. This content is distributed under the terms of the Creative Commons Attribution 4.0 International license.

### Multiple carbapenemase-producing isolates point to intrapatient plasmid dissemination.

**FIG 2:**
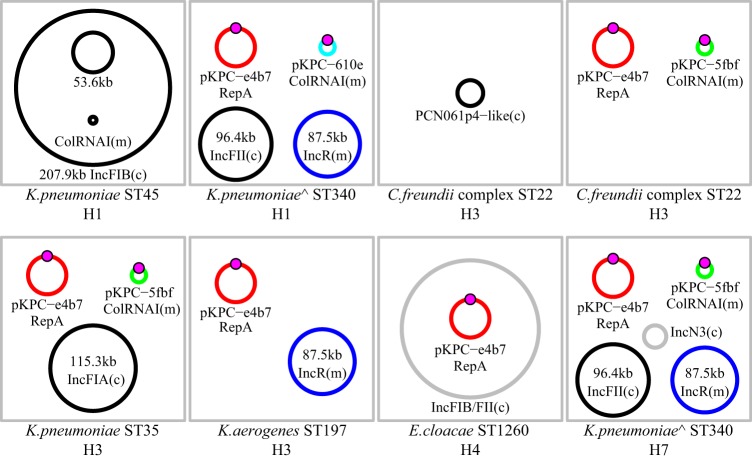
Plasmid composition of sequenced *Enterobacteriaceae*. WGS assemblies were scaffolded onto reference sequences. Plasmids shared across genomes are shown in the same color. The Tn*4401b*-KPC3 cassette is shown as a magenta circle. Plasmids that are unique to a strain are in black. Plasmids that were detected but could not be scaffolded on an existing reference sequence are shown in gray. The two *K. pneunomiae* ST340 isolates with chromosomal *bla*_KPC_ genes are marked with “^”. Hospitalization number is indicated below each isolate (H1 to H7). Plasmid incompatibility groups were determined using PlasmidFinder v2.0. Plasmid mobility was determined using MOB-suite. Conjugative plasmids are denoted with “(c),” and mobilizable plasmids are marked with “(m).” Nonmobilizable plasmids are unmarked.

The detection of carbapenem-resistance in five different bacterial strains across 4 months suggested plasmid-based horizontal gene transfer. To investigate this, the patient’s initial carbapenem-susceptible K. pneumoniae strain and six carbapenemase-producing *Enterobacteriaceae* strains collected from 2014 to 2016 were sequenced (Table 1 and Fig. 2). The four K. pneumoniae genomes belong to three different sequence types (susceptible, ST45; resistant, ST340 and ST35). Carbapenemase-producing Citrobacter freundii (ST22), *K. aerogenes* (ST197), and Enterobacter cloacae (ST1260) strains were sequenced from the third and fourth hospitalizations. Finally, a carbapenem-susceptible strain of C. freundii was also sequenced.

**TABLE 1:** Isolates sequenced in this study^*a*^

Species	ST	Source	Day post-KPC^+^	WGS	Chromosome size (Mb)	KPC	No. of plasmids	Plasmid variant of:	
								pMNCRE78_3	pNJST258C2- like
K. pneumoniae	ST45	Blood	−10 (H1)	Complete	5.3	Neg	3	None	None
	ST340	Abscess	0 (H1)	Complete	5.3	KPC3	4	pKPC-e4b7	pKPC-610e
C. freundii	ST22	Abscess	39 (H3)	Contigs	4.9	Neg	1	None	None
	ST22	Perirectal	68 (H3)	Complete	4.9	KPC3	2	pKPC-e4b7	pKPC-5fbf
K. pneumoniae	ST35	Perirectal	68 (H3)	Complete	5.3	KPC3	3	pKPC-e4b7	pKPC-5fbf
*K. aerogenes*	ST197	Perirectal	68 (H3)	Contigs	ND	KPC3	2	pKPC-e4b7	None
E. cloacae	ST1260	Perirectal	124 (H4)	Contigs	ND	KPC3	2	pKPC-e4b7	None
K. pneumoniae	ST340	Perirectal	435 (H7)	Contigs	5.3	KPC3	5	pKPC-e4b7	pKPC-5fbf

a H1 to H7 are hospital admissions. PacBio genomes are marked as “Complete,” and MiSeq assemblies are marked as “Contigs.” The plasmid compositions of draft genomes were inferred from contig scaffolding and PCR results. Neg, negative; ND, no data.

### Two plasmids are found across CPOs.

Analysis of the whole-genome sequence data from the carbapenemase-producing organisms (CPOs) in Fig. 1 determined that they all carry the KPC-3 allele in the context of a Tn*4401b* cassette on one or two plasmids. The first plasmid (pKPC-e4b7) is nearly identical to pMNCRE78_3 from a wound-associated Klebsiella pneumoniae strain (2013, Minnesota Department of Health, unpublished data [GenBank accession no. CP018432.1]) aligning over 99% of its length with only 3 single nucleotide polymorphisms (SNPs) and 2 indels (see Fig. S1 in the supplemental material). The SNPs are in a transglycosylase (g.10957C>T, p.Asn=), Tn3-family transposase (g.27840T>C, p.Phe984Leu), and in the *bla*_KPC_ gene (g.31028C>T, p.His272Tyr). The *bla*_KPC_ mutation reflects the KPC-3 allele in our isolates compared to a KPC-2 allele in the reference plasmid. Our lab and others have observed the phenomenon of essentially identical plasmids with different KPC alleles previously with pKpQIL (6, 7). An insertion (g.31_32insA) corrects the reading frame of an annotated pseudogene for a type II/IV secretion system family protein. Finally, there is a 490-nucleotide (nt) deletion in a pseudogene (g.13534_14023del). There were no additional shared SNPs that could be used to identify transmission links between bacteria.

10.1128/mBio.00652-19.1 FIG S1 (A) Alignment of pKPC-e4b7 to the reference sequence pMNCRE78_3 (CP018432.1); variants described in the text are annotated below pMNCRE78_3. (B) Alignment of the reference sequence pNJST258C2 (CP006919.1) with plasmid variants described in this study. Positions of PCR amplicons used to detect pKPC-610e and pKPC-5fbf are underlined in blue. Antimicrobial resistance genes are yellow, transposase/resolvase genes are in brown, genes with annotated functions are in green, and hypothetical genes are in gray. The Tn*4401* cassette is underlined in green. Differences between the annotations of aligned regions are largely due to differences in annotation pipelines and databases. Download FIG S1, EPS file, 1.1 MB. Copyright © 2019 Conlan et al. 2019 Conlan et al. This content is distributed under the terms of the Creative Commons Attribution 4.0 International license.

The second KPC-positive plasmid, carried by a subset of sequenced isolates, is related to pNJST258C2 (8) and occurs in two variants (pKPC-610e and pKPC-5fbf) differentiated by sequence inversions (Fig. S1). Long-read sequencing of the ST340 K. pneumoniae isolate also revealed a chromosomal copy of the *bla*_KPC-3_ gene embedded in a Tn*4401b* cassette. The ST340 K. pneumoniae and *K. aerogenes* isolates share an additional plasmid from the IncR incompatibility group (Fig. 2; blue plasmid). It carried genes for resistance to a variety of antibiotics (e.g., aminoglycosides, chloramphenicol, and macrolides), two class 1 integrons (In27 and In641) (9), and an iron transport locus.

### Detection of putative C. freundii recipient strain.

A carbapenem-susceptible C. freundii strain was identified on day 39, prior to detection of the carbapenemase-producing C. freundii strain on day 68 (Fig. 1). We sequenced the susceptible strain and determined that it was the same sequence type as the resistant isolate and that the two isolates differed by only 15 SNPs across 99.2% of the chromosome. As expected, the susceptible strain did not carry either *bla*_KPC_-containing plasmid but it did carry a plasmid with similarity to PCN061p4 (CP006640.1). The carbapenemase-producing C. freundii strain lacked the PCN061p4 plasmid but carried both pKPC-e4b7 and pKPC-5fbf, which were previously detected in other *Enterobacteriaceae*.

### *bla*_KPC_ plasmid-containing isolates persist for over a year.

Of the five carbapenemase-producing isolates sequenced from the third and fourth hospitalizations, all of them carried the *bla*_KPC_-positive pMNCRE78_3 plasmid, and two carried the *bla*_KPC_-positive pNJST258C2-like plasmid. Using PCR primers specific to each plasmid (see Materials and Methods), we found that both *bla*_KPC_-positive plasmids persisted through the seventh hospitalization, 435 days after the first *bla*_KPC_-positive culture (Fig. 1). Of the 10 isolates tested from the sixth and seventh hospitalizations, seven carried the pMNCRE78_3 plasmid and nine carried one of the pNJST258C2 plasmid variants. An ST340 K. pneumoniae isolate from day 435 was sequenced, found to be nearly identical (<10 chromosomal SNPs) to the initial ST340 isolate, and carried all four plasmids. However, it now carries the pNJST258C2-like plasmid with sequence inversions and an additional small IncN3 plasmid (Fig. 2).

### Plasmid mobility.

Plasmids may move between bacterial hosts by a number of mechanisms, but conjugation and mobilization (MOB) are the most relevant in the current context. Conjugation requires genes for mating pair formation (MPF) and mobilization (MOB), while mobilizable plasmids only require MOB genes and are thought to use MPF systems encoded by other plasmids (10). We used MOB-suite (11) and PlasmidFinder (12) to characterize all the replicon type and predicted mobility of all 12 plasmids in this study. pKPC-e4b7 and the reference pMNCRE78_3 plasmid are both predicted to be nonmobilizable, despite being annotated to encode some genes associated with a type IV secretion system. pKPC-610e and pKPC-5fbf are predicted to be mobilizable, since they carry a MOBC type relaxase gene. Overall, of the 12 unique plasmid types in this study, six are predicted to be conjugative, 4 are mobilizable, and 2 are nonmobilizable (Fig. 2).

### Antibiotics alter the gut microbiome and select for a drug-resistant strain.

In previous studies of plasmid carriage in patients (13), we have noted that culture-based studies are limited in that single isolates are a nonrepresentative subsample of the patient microbiome. While we have been successful in using culture-enriched metagenomes (i.e., “plate swipes”) to characterize microbial communities (14), here we sought to explore the utility of unbiased fully metagenomic sequencing to characterize the changes in the microbiome associated with a small window of time immediately following administration of a course of meropenem.

Stool and perirectal swabs were collected at three time points during March through April 2015 (T1, T2, and T3 [Fig. 1]) during the patient’s third hospitalization and were subjected to shotgun sequencing to capture the metagenome. Immediately following enrollment in the study, baseline samples were taken from stool and perirectal swabs on the same day as a 4-day course of meropenem and a single-day course of gentamicin were initiated (T1). One day later, the patient began a 13-day course of levofloxacin. The patient also received an extended 42-day course of daptomycin, initiated the previous month. Additional perirectal and stool samples were acquired approximately 1 week (T2) and 2 weeks (T3) after T1. While somewhat complicated by previous antibiotic exposures and the patient’s multiple overwhelming infections, these samples provide an opportunity to examine the response of the microbiome and target organisms to the stress of combination antibiotic therapy.

**FIG 3:**
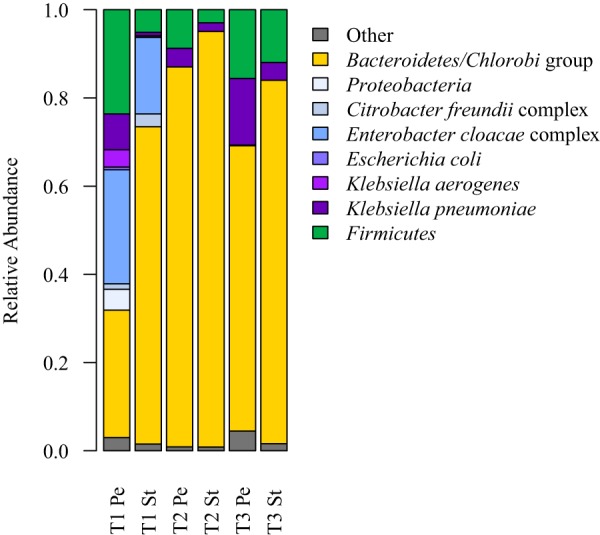
Metagenomic read classification of bacteria. *Proteobacteria*, particularly *Enterobacteriaceae*, are shown in shades of blue and purple. Relative abundances have been normalized by genome length. Pe, perirectal swab; St, stool. Time points are labeled T1 to T3.

We calculated the relative abundances of bacterial taxa, with special emphasis on taxa matching cultured isolates (Fig. 3). As expected for gut-associated communities, all samples were dominated by *Bacteroidetes*, *Firmicutes*, and *Proteobacteria*. The *Proteobacteria* present were predominantly (>98%) *Gammaproteobacteria*, and specifically *Enterobacteriaceae*. At T1, the *Enterobacteriaceae* were a mixture of *Klebsiella*, *Citrobacter*, *Enterobacter*, and *Escherichia* species. After the administration of meropenem, gentamicin, and levofloxacin, K. pneumoniae became the dominant *Enterobacteriaceae* species (T2 and T3).

The loss of *Enterobacteriaceae* diversity may be explained by the antibiotic resistance profiles of each strain. Specifically, the ST340 K. pneumoniae strain is resistant to levofloxacin, which was given immediately after T1. The resistance of this strain to quinolones, like levofloxacin, is likely due to mutations in *gyrA* (D87Y) and *parC* (S80I) (15). The ST340 sequence type is part of the multidrug-resistant clonal complex 258, and other ST340 reference strains also have the drug-resistant *gyrA* allele (e.g., CAV1217 [CP018676.1] and CAV1417 [CP018352.1]). Read mapping to the *gyrA* gene showed 100% susceptible *gyrA* alleles at T1 (24 reads supporting) and 100% resistant alleles at T2 and T3 (22 reads supporting each). Similar results were seen for the S80I mutation in *parC*, with 30/31 reads supporting susceptibility at T1 and 100% resistant alleles at T2 and T3 (10 and 6 reads, respectively). The ST340 strain carried additional resistance genes for aminoglycosides: ANT(3″)-Ia family aminoglycoside nucleotidyltransferase genes *aadA1* and *aadA2*, as well as aminoglycoside *O*-phosphotransferase APH(3′)-Ia, macrolide resistance, and chloramphenicol resistance genes.

## DISCUSSION

HSCT offers patients with hematologic malignancies the chance for a cure or remission. However, conditioning regimens, long-term immunosuppressive therapy, and frequent hospitalization are all risk factors that complicate treatment. We investigated the impact of HSCT and the follow-up treatments and therapies on the patient’s microbiome and several target organisms with pathogenic potential. Because of the patient’s fragile medical condition and colonization with organisms of infection control concern, we conducted close clinical and microbiological monitoring that made it possible to detect the HGT. Despite infections with multidrug-resistant organisms and severe immunosuppression, the patient ultimately recovered, was discharged home, and continues to return for clinic visits.

Multidrug-resistant bacteria are an unavoidable consequence of decades of antibiotic use. As such, combination therapies are commonly used to both prevent and treat bacterial infections in vulnerable patients. In the current case, treatment with multiple classes of antibiotics following the detection of a single carbapenemase-producing K. pneumoniae strain resulted in the detection of multiple carbapenemase-producing organisms, including *Citrobacter* and *Enterobacter* spp. While we cannot rule out that additional resistance mechanisms may be at play (e.g., efflux pumps or outer membrane protein mutations), we did not detect disruptive mutations in the K. pneumoniae OmpK35/36 porins, which are known to be associated with carbapenem resistance (16). Furthermore, the presence of two plasmids carrying the *bla*_KPC_ gene makes it likely that HGT underlies the rapid spread of carbapenem resistance. While HGT within the gut of the patient is one possible explanation of these findings, without additional studies we cannot rule out other hypotheses. Limited dissemination of plasmids *in vivo* has been described previously in patients (13, 17–19) and in a mouse model (20)—however, not to the extent seen in the present study with two plasmids distributed across five strains. Stecher and colleagues describe high rates of HGT during blooms of *Enterobacteriaceae* in a mouse model of *Salmonella* infection (21).

Plasmids can be assigned to three mobility groups: conjugative, mobilizable, and nonmobilizable. Surprisingly, a prevalent *bla*_KPC_ plasmid (pKPC-e4b7) associated with multiple species isolated from this patient is predicted to be nonmobilizable. While it is possible that pKPC-e4b7 carries a relaxase (*mob*) gene that is not detected by the MOB-suite analysis, we did not detect one using additional blastp/blastx searches against a database of Mob proteins. Other mechanisms of HGT like transformation and transduction are predicted to be low-frequency events that would not explain this plasmid’s broad distribution. Despite this, it is clear that pKPC-e4b7 is present in five different hosts isolated over a relatively short period of time.

One limitation of this work is that we did not demonstrate *in vitro* plasmid transfer. Previously, we have shown that *in vitro* plasmid conjugation experiments often do not recapitulate what is seen *in vivo* (22), and we expect that will be true in this case. That said, several lines of evidence support recent dissemination of these plasmids, possibly in the patient. The pKPC-e4b7 plasmid is essentially identical across strains, supporting recent transmission events. We routinely sequence all *bla*_KPC_-positive isolates from the NIH Clinical Center, and we have not observed this plasmid in patients or the environment (7, 23), suggesting it is not an endemic plasmid. Finally, we identified the probable recipient *Citrobacter* strain in the patient prior to the detection of the plasmid-containing carbapenemase-producing strain.

We investigated the changes in the patient’s microbiome using shotgun metagenomics. Stool and perirectal surveillance swabs provided complementary and mostly correlative measures of microbial relative abundance. That said, metagenomes prepared from perirectal swabs were dominated by host-derived reads, making them a suboptimal choice for characterizing the bacterial component of the microbiome. Furthermore, in cases such as this one where target bacteria are easily cultivated, we would recommend the plate swipe method (14) for assessing bacterial diversity. Unfortunately, that was not possible in the current study because the primary samples were frozen immediately upon collection.

The bacterial component of the microbiome showed a number of changes as a result of antibiotic therapy. Specifically, there was a reduction in the diversity of *Enterobacteriaceae*, with Klebsiella pneumoniae becoming the dominant species. The dominance of K. pneumoniae was driven by the clonal expansion of a single multidrug-resistant ST340 strain, part of clonal complex 258. In particular, the apparent fixation of fluoroquinolone-resistant alleles highlights the potential consequences of multidrug antibiotic therapies. All inpatients in our hospital undergo screening for carbapenemase-producing organisms. However, this patient’s higher clinical acuity exposed the patient to a greater intensity of microbial scrutiny, which may have made the HGT more apparent by greater sampling. In addition, the patient’s immunosuppressive therapy as well as effects of antimicrobial pressure from treatment of multiple concurrent multidrug-resistant infections may have played roles in these findings.

## MATERIALS AND METHODS

### Sample collection and bacterial culturing.

The patient provided written consent to participate in a study approved by the institutional review board of the National Institutes of Health Clinical Center (https://clinicaltrials.gov/ct2/show/NCT01933620). Perirectal surveillance cultures were plated directly onto CRE Chromagar (Hardy Diagnostics) and incubated overnight at 35°C in ambient air. Blood cultures were collected into Bactec Plus bottles (Becton, Dickinson). Other clinical cultures were plated onto routine clinical microbiology media, which included a combination of sheep blood agar (Remel, Inc.), chocolate agar (Remel, Inc.), MacConkey agar (Remel, Inc.), and/or fastidious broth (Hardy Diagnostics) and incubated at 35°C in 5% CO_2_. Organisms were identified by matrix-assisted laser desorption ionization–time of flight mass spectrometry (MALDI-TOF MS) (BioTyper; Bruker, Billerica, MA). Susceptibility testing was performed via broth microdilution (Trek Diagnostics) or disk diffusion (Thermo Fisher Scientific, Lenexa, KS) following Clinical and Laboratory Standards Institute M100 interpretive breakpoints that were current for the time (24–26). Isolates meeting internal laboratory testing criteria were assessed by real-time PCR for the presence of *bla*_KPC_ or *bla*_NDM_ (http://www.cdc.gov/HAI/pdfs/labSettings/KPC-NDM-protocol-2011.pdf). Primary stool and perirectal samples were frozen at –80°C for subsequent DNA extraction and metagenomic analyses.

### Genome sequencing and assembly.

Genomic DNA was purified for each isolate, from which Nextera XT (Illumina) libraries were generated. Each isolate was sequenced using a paired-end 300-base dual index run on an Illumina MiSeq. The reads were subsampled to achieve 80× coverage and then assembled with SPAdes (version 3.6.0) (27) and polished using Pilon (28). To achieve full reference genomes for select isolates, genomic DNA was sequenced on PacBio RSII SMRT platform (P6 polymerase binding and C4 sequencing kit; 240-min collection), and assembled and polished using Canu v1.3 (29). Genome annotation was performed using National Center for Biotechnology Information (NCBI) Prokaryotic Genome Annotation Pipeline (PGAP [https://www.ncbi.nlm.nih.gov/genome/annotation_prok/]). The multilocus sequence type (MLST) databases at https://pubmlst.org (30) were used to sequence type isolates; *K. aerogenes* ST197 and E. cloacae ST1260 were identified in this study. Whole-genome shotgun assemblies were scaffolded on existing references using ABACAS (31) and the mummer package (32). Plasmid incompatibility groups were predicted using PlasmidFinder 2.0 (12), and plasmid mobility was predicted using MOB-suite (11).

### Metagenome analysis.

Metagenomic samples were processed and analyzed as described previously (33, 34). Briefly, Nextera XT library kits were used to generate Illumina libraries per the manufacturer’s instructions. Libraries were sequenced on an Illumina HiSeq at the NIH Intramural Sequencing Center to a target depth of 15 to 50 million clusters of 2 × 125-bp reads. Human reads were removed based on alignment to the human reference genome (hg19) using bowtie2, bases with quality scores below 20 were trimmed, and remaining reads less than 50 bp were removed. Reads were assigned to a microbial reference genome database of bacteria, fungi, viruses, and archaea using Clinical Pathoscope as described previously (34, 35).

### Primer design.

Primers were designed using Primer3 (36) for the pMNCRE78_3 plasmid (CP018432.1) in the BTE52_30285 (Tn) and BTE52_30260 (hypothetical) genes. Primers were designed for the pNJST258C2 plasmid (CP006919) in the KPNJ2_05469 (hypothetical) gene and flanking one of the recombination junctions (see Table S2 and Fig. S1 in the supplemental material).

10.1128/mBio.00652-19.4 TABLE S2 Primers used in this study. Download Table S2, DOCX file, 0.1 MB. Copyright © 2019 Conlan et al. 2019 Conlan et al. This content is distributed under the terms of the Creative Commons Attribution 4.0 International license.

### Accession number(s).

GenBank accession numbers for previously published plasmids are included in the text (e.g., CP018432.1, CP006919.1, and CP006640.1). Sequences determined in this work have been deposited under GenBank accession no. CP036442 to CP036445, CP036446 to CP036450, CP036438 to CP036441, CP036435 to CP036437, SJEU00000000, PQLE00000000, SKFJ00000000, and SJEH00000000.

10.1128/mBio.00652-19.2 FIG S2 Multikingdom metagenomic read classification. *Proteobacteria*, particularly *Enterobacteriaceae*, are shown in shades of green. Viruses are shown in shades of purple. Relative abundances have been normalized by genome length. Pe, perirectal swab; St, stool. Time points are labeled T1 to T3. Download FIG S2, EPS file, 0.8 MB. Copyright © 2019 Conlan et al. 2019 Conlan et al. This content is distributed under the terms of the Creative Commons Attribution 4.0 International license.
