# Supplementary material for: Plasmid Dissemination and Selection of a Multidrug-Resistant Klebsiella pneumoniae Strain during Transplant-Associated Antibiotic Therapy
Source: mBio. 2019 Oct 8;10(5):e00652-19. doi: 10.1128/mBio.00652-19 (PMC6786864; doi:10.1128/mBio.00652-19)
Supplement: TABLE S2 [file mBio.00652-19-st002.docx]

| **Primer** | **Purpose** | **Forward** | **Reverse** |
| --- | --- | --- | --- |
| pMN_30260 | marker gene, pMNCRE78_3 | 5’-ACGACATTCGTCAACTGCAA-3’ | 5’-ACGCACGGAACTCTATACCG-3’ |
| pMN_Tn1 | marker gene, pMNCRE78_3 | 5’-CCCCAGACAAAGGTTTTGAA-3’ | 5’-CCCTTCATTTCCTGTCGGTA-3’ |
| pNJ_05469 | marker gene, pNJST258C2 | 5’-ATTCCGTTTCAGCCGTCAAG-3’ | 5’-ACACCACTTCCTGATACCGG-3’ |
| pNJ_Cjun | junction (pKPC-610e) | 5’-CCACAGCGCCTCAGATAGA-3’ | 5’-AGGCAATCCCGAACAGGTTA-3’ |
| pNJ_Kjun | junction (pKPC-5fbf) | 5’-CCACAGCGCCTCAGATAGA-3’ | 5’-TCCATGAGACTGCTGGAACA-3’ |
| CRE28 | KPC primers | 5’-CAGCTCATTCAAGGGCTTTC-3’ | 5’-GGCGGCGTTATCACTGTATT-3’ |

**TABLE S1** Primers used in this study
